# (*S*)-2-[(*S*)-2,2,2-Tri­fluoro-1-hy­droxy­eth­yl]-1-tetra­lone

**DOI:** 10.1107/S2414314622012093

**Published:** 2023-01-06

**Authors:** Klemen Motaln, Andrej Emanuel Cotman, Matic Lozinšek

**Affiliations:** a Department of Inorganic Chemistry and Technology, Jožef Stefan Institute, Jamova cesta 39, 1000 Ljubljana, Slovenia; b Jožef Stefan International Postgraduate School, Jamova cesta 39, 1000 Ljubljana, Slovenia; c Department of Pharmaceutical Chemistry, Faculty of Pharmacy, University of Ljubljana, Aškerčeva cesta 7, 1000 Ljubljana, Slovenia; Vienna University of Technology, Austria

**Keywords:** tetra­lone, crystal structure, asymmetric catalysis, O—H⋯O hydrogen bonding

## Abstract

The crystal structure of the title compound, C_12_H_11_F_3_O_2_, was elucidated by low-temperature single-crystal X-ray diffraction. The enanti­opure compound crystallizes in the Sohncke space group *P*2_1_ and features one mol­ecule in the asymmetric unit. The structure displays inter­molecular O—H⋯O hydrogen bonding, which links the mol­ecules into infinite chains propagating parallel to [010]. The absolute configuration was established from anomalous dispersion.

## Abstract 2

The title tetra­lone derivative crystallizes in the Sohncke space group *P*2_1_ and features one mol­ecule in the asymmetric unit. In the crystal, mol­ecules are hydrogen-bonded into infinite zigzag chains running parallel to [010].

## Graphical abstract

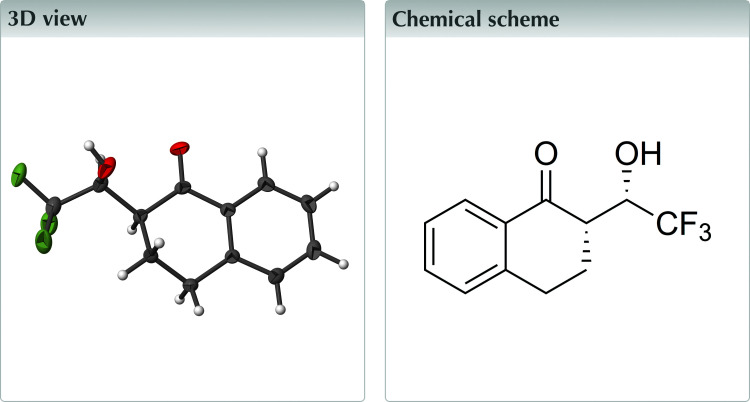

## Structure description

**Figure 1:**
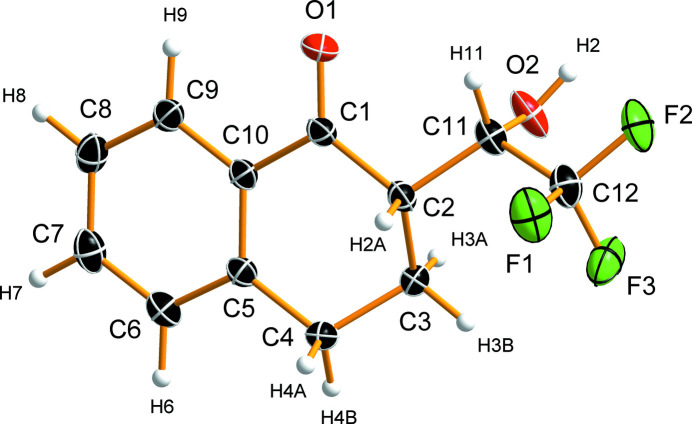
Mol­ecular structure of the title compound and the atom-labeling scheme. Displacement ellipsoids are shown at the 50% probability level and hydrogen atoms are depicted as spheres of arbitrary radius.

Dynamic kinetic resolution (DKR) based on Ru^II^-catalyzed Noyori–Ikariya asymmetric transfer hydrogenation (ATH) has proven to be a highly efficient strategy for the stereoconvergent synthesis of secondary alcohols (Cotman, 2021 ▸). The commercial availability of a wide range of Ru^II^ catalysts, comparatively mild reaction conditions, and the ability to use racemic mixtures of ketones as starting materials make this approach particularly attractive for the synthesis of β-substituted benzyl alcohols, which have been shown to be valuable building blocks for pharmaceuticals and can crystallize as homochiral single-component mechanically responsive crystals that exhibit elastic or plastic flexibility (Cotman *et al.*, 2019 ▸, 2022 ▸). When ATH of non-symmetric CF_3_-substituted 1,3-diketones was attempted, it was found that two consecutive DKR–ATH reactions can occur and that diastereo- and enanti­opure 1,3-diols can be obtained in a one-pot process (Cotman *et al.*, 2016 ▸). The use of milder reaction conditions enabled the preparation of mono-reduced alcohols, which include the title compound.

(*S*)-2-[(*S*)-2,2,2-tri­fluoro-1-hy­droxy­eth­yl]-1-tetra­lone crystallizes in the monoclinic space group *P*2_1_ with one mol­ecule in the asymmetric unit (Fig. 1 ▸). The cyclo­hexa­none ring adopts a half-boat (envelope) conformation (Cremer & Pople, 1975 ▸), with atoms C1, C2, C4, C5, and C10 being essentially coplanar (r.m.s.d. of 0.007 Å), whereas the C3 atom is located 0.683 (2) Å below this plane. Moreover, the atoms of the planar part of the cyclo­hexa­none ring are essentially coplanar with the aromatic ring. The dihedral angle between the planes (plane normals) is 2.01 (6)° and the r.m.s.d. of the plane defined by atoms C1, C2, C4–C10 is 0.019 Å. A similar half-boat conformation was previously observed in the structure of (±)-1-tetra­lone-3-carb­oxy­lic acid (CSD refcode QIJGAR), whereas the related (±)-1-tetra­lone-2-acetic acid (QIJGEV) exhibits a half-chair conformation (Barcon *et al.*, 2001 ▸).

**Figure 2:**
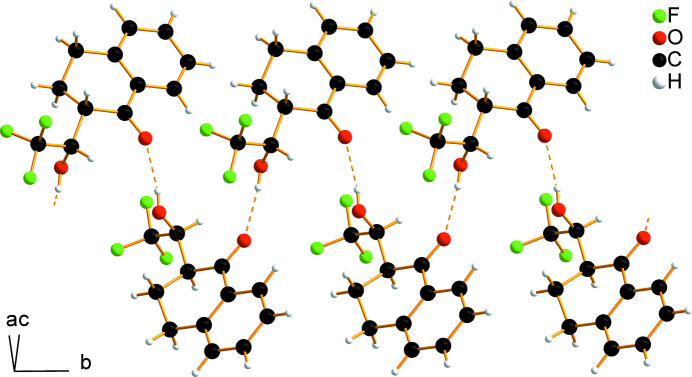
Inter­molecular O—H⋯O=C hydrogen bonds connect the mol­ecules into infinite zigzag chains running parallel to [010].

In the crystal structure of the title compound, inter­molecular O—H⋯O hydrogen bonds with an O⋯O distance of 2.7548 (16) Å (Table 1 ▸), involving hydroxyl and carbonyl groups of the adjacent mol­ecules related by the 2_1_ screw axis, link the mol­ecules into infinite zigzag chains propagating parallel to [010] (Figs. 2 ▸, 3 ▸). The graph-set motif of the chains is *C*(6) (Etter *et al.*, 1990 ▸).

## Synthesis and crystallization

The title compound was prepared from 2-tri­fluoro­acetyl-1-tetra­lone (242 mg, 1.0 mmol) added to a HCO_2_H/Et_3_N 5:2 (0.5 ml) solution containing the active (*S*,*S*)-di­phenyl­ethyl­enedi­amine-based Ru^II^ catalyst with an S:C ratio of 2000:1 (Cotman *et al.*, 2016 ▸). Upon addition of the co-solvent chloro­benzene (1 ml), the mixture was warmed to 40 °C and stirred for 23 h, while being continuously flushed with N_2_. The resulting mixture was partitioned between EtOAc (10 ml) and H_2_O (5 ml), with the organic layer later washed with H_2_O (5 ml) and brine (5 ml), filtered through a bed of silica gel/Na_2_SO_4_, and concentrated. The procedure resulted in the formation of a crude white product (239 mg, 98% yield), containing the title compound (d.r. = 89:11, 72% ee) and 2.5% of the corresponding diol. After purification by flash chromatography (hexa­ne/EtOAc gradient 9:1 to 7:1), the diastereomerically pure monoalcohol was isolated (157 mg, 64% yield). The enanti­omeric excess was upgraded to >99% by crystallization from cyclo­hexane (109 mg, 45% yield). Crystals suitable for single-crystal X-ray diffraction analysis were grown from a chloro­form solution. A suitable crystal was selected under a polarizing microscope and mounted on a MiTeGen Dual Thickness MicroLoop LD using Baysilone-Paste (Bayer-Silicone, mittelviskos).

## Refinement

Crystal data, data collection, and structure refinement details are summarized in Table 2 ▸. The positions of the hydrogen atoms were freely refined, including their isotropic displacement parameter *U* (Cooper *et al.*, 2010 ▸). The absolute configuration was established as *S*,*S* for C2 and C11, respectively, based on the anomalous dispersion effects [Flack *x* = −0.07 (3); Hooft *y* = −0.04 (2); Parsons *et al.*, 2013 ▸; Hooft *et al.*, 2008 ▸].

## Supplementary Material

Crystal structure: contains datablock(s) I. DOI: 10.1107/S2414314622012093/wm4178sup1.cif

Structure factors: contains datablock(s) I. DOI: 10.1107/S2414314622012093/wm4178Isup2.hkl

Supporting information file. DOI: 10.1107/S2414314622012093/wm4178Isup3.cml

CCDC reference: 2232401

Additional supporting information:  crystallographic information; 3D view; checkCIF report

## Figures and Tables

**Figure 3:**
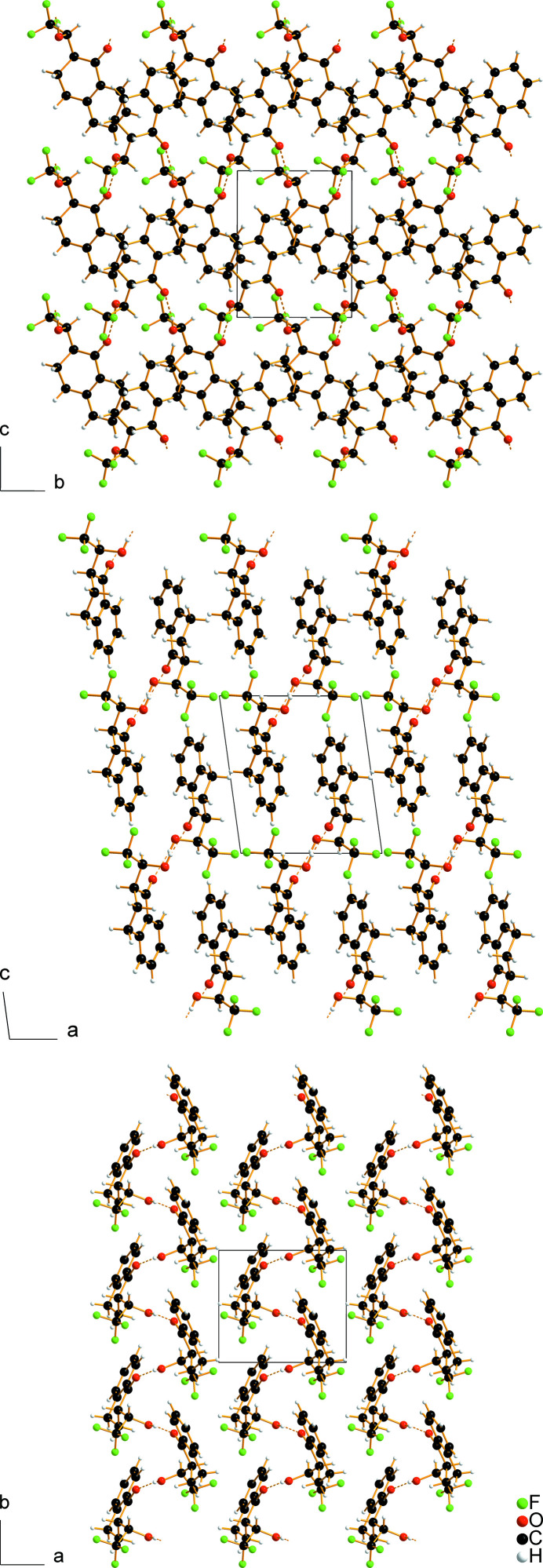
Packing diagrams of the title compound viewed along [100] (top), [010] (middle), and [001] (bottom).

**Table 1:** Hydrogen-bond geometry (Å, °)

*D*—H⋯*A*	*D*—H	H⋯*A*	*D*⋯*A*	*D*—H⋯*A*
O2—H2⋯O1^i^	0.79 (3)	1.97 (3)	2.7548 (16)	168 (3)

Symmetry code: (i) 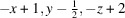 .

**Table 2:** Experimental details

Crystal data	
Chemical formula	C_12_H_11_F_3_O_2_
*M*_r_	244.21
Crystal system, space group	Monoclinic, *P*2_1_
Temperature (K)	100
*a*, *b*, *c* (Å)	8.23147 (11), 7.16385 (9), 9.24494 (14)
β (°)	97.8459 (13)
*V* (Å^3^)	540.06 (1)
*Z*	2
Radiation type	Cu *K*α
μ (mm^−1^)	1.18
Crystal size (mm)	0.16 × 0.10 × 0.07

Data collection	
Diffractometer	XtaLAB Synergy-S, Dualflex, Eiger2 R CdTe 1M
Absorption correction	Gaussian (*CrysAlis PRO*; Rigaku OD, 2022 ▸)
*T*_min_, *T*_max_	0.832, 1.000
No. of measured, independent and observed [*I* > 2σ(*I*)] reflections	13577, 2210, 2186
*R*_int_	0.025
(sin θ/λ)_max_ (Å^−1^)	0.629

Refinement	
*R*[*F* ^2^ > 2σ(*F* ^2^)], *wR*(*F* ^2^), *S*	0.022, 0.056, 1.06
No. of reflections	2210
No. of parameters	198
No. of restraints	1
H-atom treatment	All H-atom parameters refined
Δρ_max_, Δρ_min_ (e Å^−3^)	0.14, −0.14
Absolute structure	Flack *x* determined using 976 quotients [(*I* ^+^)−(*I* ^−^)]/[(*I* ^+^)+(*I* ^−^)] (Parsons *et al.*, 2013 ▸)
Absolute structure parameter	−0.07 (3)

Computer programs: *CrysAlis PRO* (Rigaku OD, 2022 ▸), *SUPERFLIP* (Palatinus & Chapuis, 2007 ▸; Palatinus & van der Lee, 2008 ▸; Palatinus *et al.*, 2012 ▸), *SHELXL* (Sheldrick, 2015 ▸), *OLEX2* (Dolomanov *et al.*, 2009 ▸), *DIAMOND* (Brandenburg, 2005 ▸), and *publCIF* (Westrip, 2010 ▸).

## full crystallographic data

### Crystal data

**Table:**

C_12_H_11_F_3_O_2_	*F*(000) = 252
*M**_r_* = 244.21	*D*_x_ = 1.502 Mg m^−^^3^
Monoclinic, *P*2_1_	Cu *K*α radiation, λ = 1.54184 Å
*a* = 8.23147 (11) Å	Cell parameters from 11151 reflections
*b* = 7.16385 (9) Å	θ = 4.8–75.9°
*c* = 9.24494 (14) Å	µ = 1.18 mm^−^^1^
β = 97.8459 (13)°	*T* = 100 K
*V* = 540.06 (1) Å^3^	Irregular, colourless
*Z* = 2	0.16 × 0.10 × 0.07 mm

### Data collection

**Table:**

XtaLAB Synergy-S, Dualflex, Eiger2 R CdTe 1M diffractometer	2210 independent reflections
Radiation source: micro-focus sealed X-ray tube, PhotonJet (Cu) X-ray Source	2186 reflections with *I* > 2σ(*I*)
Mirror monochromator	*R*_int_ = 0.025
Detector resolution: 13.3333 pixels mm^-1^	θ_max_ = 76.0°, θ_min_ = 4.8°
ω scans	*h* = −10→10
Absorption correction: gaussian (CrysAlisPro; Rigaku OD, 2022)	*k* = −9→8
*T*_min_ = 0.832, *T*_max_ = 1.000	*l* = −11→11
13577 measured reflections	

### Refinement

**Table:**

Refinement on *F*^2^	Hydrogen site location: difference Fourier map
Least-squares matrix: full	All H-atom parameters refined
*R*[*F*^2^ > 2σ(*F*^2^)] = 0.022	*w* = 1/[σ^2^(*F*_o_^2^) + (0.0312*P*)^2^ + 0.0761*P*] where *P* = (*F*_o_^2^ + 2*F*_c_^2^)/3
*wR*(*F*^2^) = 0.056	(Δ/σ)_max_ < 0.001
*S* = 1.06	Δρ_max_ = 0.14 e Å^−^^3^
2210 reflections	Δρ_min_ = −0.14 e Å^−^^3^
198 parameters	Absolute structure: Flack *x* determined using 976 quotients [(*I*^+^)–(*I*^–^)]/[(*I*^+^)+(*I*^–^)] (Parsons *et al.*, 2013)
1 restraint	Absolute structure parameter: −0.07 (3)
Primary atom site location: iterative	

### Special details

**Table:**

Geometry. All esds (except the esd in the dihedral angle between two l.s. planes) are estimated using the full covariance matrix. Standard uncertainties involving l.s. planes were estimated using ShelXL matrix (within Olex2). The cell esds are taken into account individually in the estimation of esds in distances, angles and torsion angles; correlations between esds in cell parameters are only used when they are defined by crystal symmetry. An approximate (isotropic) treatment of cell esds is used for estimating esds involving l.s. planes.

### Fractional atomic coordinates and isotropic or equivalent isotropic displacement parameters (Å^2^)

**Table:**

	*x*	*y*	*z*	*U*_iso_*/*U*_eq_	
F2	0.26550 (14)	0.33275 (16)	1.13591 (11)	0.0382 (3)	
F1	0.04271 (12)	0.42167 (17)	1.00572 (12)	0.0399 (3)	
F3	0.18733 (16)	0.20130 (15)	0.92934 (12)	0.0410 (3)	
O1	0.35100 (16)	0.86561 (17)	0.83139 (13)	0.0334 (3)	
O2	0.44946 (14)	0.4422 (2)	0.91134 (13)	0.0332 (3)	
C10	0.27986 (17)	0.8069 (2)	0.58052 (17)	0.0193 (3)	
C1	0.28808 (18)	0.7590 (2)	0.73748 (16)	0.0213 (3)	
C5	0.20219 (17)	0.6892 (2)	0.47174 (16)	0.0189 (3)	
C2	0.21111 (17)	0.5750 (2)	0.77698 (16)	0.0201 (3)	
C9	0.34870 (17)	0.9768 (2)	0.54318 (18)	0.0239 (3)	
C6	0.19256 (18)	0.7460 (2)	0.32605 (17)	0.0240 (3)	
C12	0.1966 (2)	0.3660 (2)	0.99739 (17)	0.0276 (3)	
C3	0.21572 (18)	0.4307 (2)	0.65536 (16)	0.0209 (3)	
C11	0.29360 (18)	0.5124 (2)	0.92764 (16)	0.0239 (3)	
C8	0.33780 (18)	1.0304 (2)	0.3984 (2)	0.0279 (3)	
C4	0.12924 (17)	0.5066 (2)	0.51106 (16)	0.0219 (3)	
C7	0.25885 (19)	0.9149 (3)	0.28981 (18)	0.0277 (3)	
H2A	0.099 (2)	0.599 (3)	0.786 (2)	0.020 (4)*	
H3A	0.332 (2)	0.396 (3)	0.6443 (19)	0.019 (4)*	
H4B	0.140 (2)	0.413 (3)	0.432 (2)	0.024 (4)*	
H11	0.298 (2)	0.619 (3)	0.993 (2)	0.024 (5)*	
H9	0.406 (2)	1.054 (3)	0.620 (2)	0.028 (5)*	
H4A	0.010 (2)	0.524 (3)	0.519 (2)	0.020 (4)*	
H3B	0.165 (2)	0.314 (3)	0.680 (2)	0.030 (5)*	
H6	0.138 (2)	0.665 (3)	0.247 (2)	0.026 (5)*	
H7	0.253 (2)	0.948 (3)	0.188 (2)	0.034 (5)*	
H8	0.385 (3)	1.146 (3)	0.374 (2)	0.033 (5)*	
H2	0.501 (3)	0.433 (4)	0.990 (3)	0.051 (7)*	

### Atomic displacement parameters (Å^2^)

**Table:**

	*U*^11^	*U*^22^	*U*^33^	*U*^12^	*U*^13^	*U*^23^
F2	0.0466 (6)	0.0452 (6)	0.0231 (5)	0.0074 (5)	0.0064 (4)	0.0138 (4)
F1	0.0298 (5)	0.0523 (7)	0.0399 (6)	0.0069 (5)	0.0137 (4)	0.0133 (5)
F3	0.0648 (7)	0.0252 (5)	0.0356 (6)	−0.0015 (5)	0.0162 (5)	0.0049 (4)
O1	0.0438 (7)	0.0273 (6)	0.0248 (5)	−0.0048 (5)	−0.0104 (5)	−0.0034 (5)
O2	0.0229 (5)	0.0555 (8)	0.0204 (5)	0.0102 (5)	0.0001 (4)	0.0107 (5)
C10	0.0154 (6)	0.0193 (7)	0.0221 (7)	0.0025 (5)	−0.0008 (5)	0.0012 (5)
C1	0.0201 (6)	0.0207 (7)	0.0212 (7)	0.0022 (5)	−0.0039 (5)	−0.0006 (6)
C5	0.0156 (6)	0.0211 (7)	0.0197 (7)	0.0016 (5)	0.0011 (5)	0.0002 (5)
C2	0.0189 (7)	0.0222 (7)	0.0184 (7)	0.0012 (6)	−0.0007 (5)	0.0025 (6)
C9	0.0179 (6)	0.0201 (7)	0.0322 (8)	0.0009 (5)	−0.0017 (6)	0.0021 (6)
C6	0.0219 (7)	0.0296 (8)	0.0206 (7)	0.0026 (6)	0.0028 (5)	0.0005 (6)
C12	0.0304 (8)	0.0305 (9)	0.0226 (7)	0.0066 (6)	0.0064 (6)	0.0045 (6)
C3	0.0232 (7)	0.0173 (6)	0.0216 (7)	−0.0021 (6)	0.0009 (5)	0.0017 (6)
C11	0.0236 (7)	0.0290 (8)	0.0186 (7)	0.0038 (6)	0.0012 (5)	0.0032 (6)
C8	0.0202 (6)	0.0260 (8)	0.0377 (9)	−0.0009 (6)	0.0041 (6)	0.0093 (7)
C4	0.0233 (7)	0.0211 (7)	0.0200 (7)	−0.0037 (6)	−0.0011 (5)	−0.0009 (6)
C7	0.0244 (7)	0.0339 (8)	0.0257 (8)	0.0048 (7)	0.0072 (6)	0.0086 (7)

### Geometric parameters (Å, º)

**Table:**

F2—C12	1.3490 (18)	C9—C8	1.383 (2)
F1—C12	1.3405 (19)	C9—H9	0.97 (2)
F3—C12	1.335 (2)	C6—C7	1.387 (2)
O1—C1	1.2176 (19)	C6—H6	0.99 (2)
O2—C11	1.4053 (18)	C12—C11	1.514 (2)
O2—H2	0.79 (3)	C3—C4	1.5241 (19)
C10—C1	1.484 (2)	C3—H3A	1.006 (18)
C10—C5	1.398 (2)	C3—H3B	0.97 (2)
C10—C9	1.405 (2)	C11—H11	0.98 (2)
C1—C2	1.528 (2)	C8—C7	1.392 (3)
C5—C6	1.399 (2)	C8—H8	0.95 (2)
C5—C4	1.505 (2)	C4—H4B	1.00 (2)
C2—C3	1.531 (2)	C4—H4A	1.005 (19)
C2—C11	1.531 (2)	C7—H7	0.96 (2)
C2—H2A	0.956 (19)		
			
C11—O2—H2	108 (2)	F3—C12—F1	107.23 (14)
C5—C10—C1	121.31 (13)	F3—C12—C11	114.29 (13)
C5—C10—C9	120.36 (14)	C2—C3—H3A	111.1 (11)
C9—C10—C1	118.31 (13)	C2—C3—H3B	110.7 (12)
O1—C1—C10	120.71 (15)	C4—C3—C2	110.27 (12)
O1—C1—C2	121.37 (14)	C4—C3—H3A	109.6 (10)
C10—C1—C2	117.90 (12)	C4—C3—H3B	110.3 (12)
C10—C5—C6	118.53 (14)	H3A—C3—H3B	104.7 (16)
C10—C5—C4	120.58 (12)	O2—C11—C2	107.78 (12)
C6—C5—C4	120.89 (13)	O2—C11—C12	109.84 (13)
C1—C2—C3	110.74 (12)	O2—C11—H11	113.2 (12)
C1—C2—C11	108.84 (12)	C2—C11—H11	108.1 (12)
C1—C2—H2A	107.6 (12)	C12—C11—C2	113.33 (12)
C3—C2—H2A	107.9 (11)	C12—C11—H11	104.8 (12)
C11—C2—C3	114.71 (12)	C9—C8—C7	119.65 (15)
C11—C2—H2A	106.8 (11)	C9—C8—H8	119.7 (13)
C10—C9—H9	118.9 (12)	C7—C8—H8	120.7 (13)
C8—C9—C10	120.21 (14)	C5—C4—C3	111.57 (12)
C8—C9—H9	120.8 (12)	C5—C4—H4B	109.4 (11)
C5—C6—H6	120.1 (12)	C5—C4—H4A	109.5 (11)
C7—C6—C5	120.92 (15)	C3—C4—H4B	108.6 (11)
C7—C6—H6	118.9 (12)	C3—C4—H4A	109.1 (11)
F2—C12—C11	110.43 (13)	H4B—C4—H4A	108.5 (15)
F1—C12—F2	106.02 (12)	C6—C7—C8	120.31 (15)
F1—C12—C11	112.07 (13)	C6—C7—H7	118.7 (13)
F3—C12—F2	106.31 (13)	C8—C7—H7	120.9 (13)
			
F2—C12—C11—O2	66.50 (16)	C1—C2—C11—O2	−75.25 (16)
F2—C12—C11—C2	−172.89 (13)	C1—C2—C11—C12	162.99 (13)
F1—C12—C11—O2	−175.54 (13)	C5—C10—C1—O1	−177.41 (14)
F1—C12—C11—C2	−54.93 (18)	C5—C10—C1—C2	0.9 (2)
F3—C12—C11—O2	−53.29 (17)	C5—C10—C9—C8	1.0 (2)
F3—C12—C11—C2	67.31 (18)	C5—C6—C7—C8	0.6 (2)
O1—C1—C2—C3	−153.04 (14)	C2—C3—C4—C5	56.14 (16)
O1—C1—C2—C11	−26.05 (19)	C9—C10—C1—O1	1.0 (2)
C10—C1—C2—C3	28.63 (17)	C9—C10—C1—C2	179.31 (12)
C10—C1—C2—C11	155.61 (13)	C9—C10—C5—C6	−1.0 (2)
C10—C5—C6—C7	0.2 (2)	C9—C10—C5—C4	179.61 (13)
C10—C5—C4—C3	−26.80 (19)	C9—C8—C7—C6	−0.5 (2)
C10—C9—C8—C7	−0.3 (2)	C6—C5—C4—C3	153.81 (14)
C1—C10—C5—C6	177.35 (14)	C3—C2—C11—O2	49.43 (17)
C1—C10—C5—C4	−2.1 (2)	C3—C2—C11—C12	−72.34 (16)
C1—C10—C9—C8	−177.34 (14)	C11—C2—C3—C4	179.52 (12)
C1—C2—C3—C4	−56.81 (15)	C4—C5—C6—C7	179.58 (13)

### Hydrogen-bond geometry (Å, º)

**Table:**

*D*—H···*A*	*D*—H	H···*A*	*D*···*A*	*D*—H···*A*
O2—H2···O1^i^	0.79 (3)	1.97 (3)	2.7548 (16)	168 (3)

Symmetry code: (i) −*x*+1, *y*−1/2, −*z*+2.

## References

[bb1] Barcon, A., Brunskill, A. P. J., Lalancette, R. A., Thompson, H. W. & Miller, A. J. (2001). *Acta Cryst.* C**57**, 325–328. 10.1107/s0108270100020783 11250596

[bb2] Brandenburg, K. (2005). *DIAMOND*. Crystal Impact GbR, Bonn, Germany.

[bb3] Cooper, R. I., Thompson, A. L. & Watkin, D. J. (2010). *J. Appl. Cryst.* **43**, 1100–1107.

[bb4] Cotman, A. E. (2021). *Chem. Eur. J.* **27**, 39–53. 10.1002/chem.202002779 32691439

[bb5] Cotman, A. E., Cahard, D. & Mohar, B. (2016). *Angew. Chem. Int. Ed.* **55**, 5294–5298. 10.1002/anie.201600812 27001134

[bb6] Cotman, A. E., Dub, P. A., Sterle, M., Lozinšek, M., Dernovšek, J., Zajec, Ž., Zega, A., Tomašič, T. & Cahard, D. (2022). *ACS Org. Inorg. Au*, **2**, 396–404. 10.1021/acsorginorgau.2c00019 PMC9542724 36217345

[bb7] Cotman, A. E., Lozinšek, M., Wang, B., Stephan, M. & Mohar, B. (2019). *Org. Lett.* **21**, 3644–3648. 10.1021/acs.orglett.9b01069 PMC6750876 31058516

[bb8] Cremer, D. & Pople, J. A. (1975). *J. Am. Chem. Soc.* **97**, 1354–1358.

[bb9] Dolomanov, O. V., Bourhis, L. J., Gildea, R. J., Howard, J. A. K. & Puschmann, H. (2009). *J. Appl. Cryst.* **42**, 339–341.

[bb10] Etter, M. C., MacDonald, J. C. & Bernstein, J. (1990). *Acta Cryst.* B**46**, 256–262. 10.1107/s0108768189012929 2344397

[bb11] Hooft, R. W. W., Straver, L. H. & Spek, A. L. (2008). *J. Appl. Cryst.* **41**, 96–103. 10.1107/S0021889807059870 PMC2467520 19461838

[bb12] Palatinus, L. & Chapuis, G. (2007). *J. Appl. Cryst.* **40**, 786–790.

[bb13] Palatinus, L., Prathapa, S. J. & van Smaalen, S. (2012). *J. Appl. Cryst.* **45**, 575–580.

[bb14] Palatinus, L. & van der Lee, A. (2008). *J. Appl. Cryst.* **41**, 975–984.

[bb15] Parsons, S., Flack, H. D. & Wagner, T. (2013). *Acta Cryst.* B**69**, 249–259. 10.1107/S2052519213010014 PMC3661305 23719469

[bb16] Rigaku OD (2022). *CrysAlis PRO*. Rigaku Corporation, Oxford, England.

[bb17] Sheldrick, G. M. (2015). *Acta Cryst.* C**71**, 3–8.

[bb18] Westrip, S. P. (2010). *J. Appl. Cryst.* **43**, 920–925.

